# The Role of Fear, Hope, Message Fatigue, and Message Shocking Value in Promoting the Public’s Understanding and Support Toward COVID-19 Wastewater Monitoring: Experimental Study

**DOI:** 10.2196/83060

**Published:** 2026-02-27

**Authors:** Moonsun Jeon, Youllee Kim

**Affiliations:** 1 Department of Communication, Michigan State University, East Lansing, MI, United States; 2 Department of Communication Studies, University of Denver, 2000 E Asbury Ave, 298 Sturm Hall, Denver, CO, 80208, United States, +1 3038712385

**Keywords:** emotional appeals, we-language vs you-language, COVID-19 preventive behaviors, message fatigue, message shocking value

## Abstract

**Background:**

Although wastewater monitoring is an effective, nonintrusive public health strategy for tracking community-level COVID-19 prevalence, there has been limited research on public perceptions of this novel surveillance method. A significant gap exists in understanding how to design effective communication campaigns to gain public support for wastewater monitoring and persuade individuals to take preventive actions based on the data.

**Objectives:**

This study aimed to examine the impact of emotional appeals (fear vs hope) and pronoun use (we vs you) on public support for COVID-19 wastewater monitoring, intentions to discuss the intervention, and intentions to engage in preventive behaviors.

**Methods:**

An online 2 (emotional appeal: fear vs hope) × 2 (pronoun use: you-language vs we-language) between-subjects experiment with a control condition was conducted. A total of 603 US residents were recruited via Prolific in July 2023. Participants were randomly assigned to view 1 of 5 message stimuli and then completed a survey measuring emotional responses, message judgments (fatigue and shocking value), and the primary outcome variables.

**Results:**

Fear appeals led to greater support and stronger intentions to engage in preventive behaviors than hope appeals, while pronoun use had no significant effect. Additionally, message fatigue and shock value mediated the relationship between the message-evoked emotions and the outcome variables, whereby message fatigue was negatively associated with the outcome variables, and message shock value was positively correlated with policy support and communication intention.

**Conclusions:**

The findings suggest that while fear-based messages are effective, communication strategies should also aim to mitigate message fatigue to sustain public engagement and support for long-term public health initiatives.

## Introduction

### Background

Since the outbreak of COVID-19, various measures have been implemented to get up-to-date information on the prevalence of infection in order to take necessary steps for the patients and to prevent the spread of the disease among community members [1,2]. One effective, nonintrusive way to track the infection is through wastewater monitoring (ie, wastewater surveillance; [3,4]). Although interest in the use of wastewater for public health surveillance has grown exponentially during the COVID-19 pandemic [5], there has been limited research on the actual perceptions and understanding of the community members regarding the novel strategy [6], including how to gain the public’s support toward this surveillance intervention and persuade them to take necessary actions based on the wastewater monitoring data.

To begin filling this gap for the campaign design, this study examines the effects of emotional appeals (fear vs hope appeal) and type of pronouns (we-language vs you-language) on support for COVID-19 wastewater monitoring, interpersonal communication about COVID-19 wastewater monitoring, and intentions for COVID-19 preventive behavior upon viewing the wastewater monitoring data. Wastewater monitoring is a relatively unfamiliar, population-level surveillance tool that relies on anonymous and indirect data, yet its success depends on public understanding and policy support [3,4]. This study uses a 2×2 experimental design to provide practical evidence for how strategic framing of emotional appeals and pronoun usage can enhance public engagement with this health surveillance technology.

We begin by reviewing the roles of fear and hope in the context of COVID-19, examining how the variations of the pronouns in fear and hope appeals may lead to different persuasive outcomes. Next, we investigate the 2 message judgments (ie, message fatigue and message shocking value) as mechanisms for how fear and hope appeals lead to persuasive outcomes. Finally, we report on and discuss a study that tests our predictions about the message effects on persuasive outcomes.

### Communicating COVID-19 Wastewater Monitoring to the Public

Wastewater surveillance involves analyzing sewage water to detect and monitor the presence and concentration of pathogens, such as viruses, shed by a community [5]. The Centers for Disease Control and Prevention launched the National Wastewater Surveillance System in September 2020 in response to the COVID-19 pandemic [7]. The National Wastewater Surveillance System coordinates with communities in the United States to detect the presence of SARS-CoV-2, which is the virus that causes COVID-19. Wastewater monitoring is an effective tool that can inform timely public health responses to prevent further spread of COVID-19, providing quick and efficient community-level data for early detection and warning [8]. Unlike other types of COVID-19 surveillance, wastewater monitoring is independent of health care access or testing availability, with the potential to detect various infectious disease threats in the future [9,10]. That is, it retains clear relevance as a long-term public health strategy because the infrastructure and analytic capacity developed during the pandemic can be leveraged to monitor a range of emerging and reemerging infectious diseases, including influenza, respiratory syncytial virus (RSV), and potential future pathogens of concern [11].

Although wastewater monitoring proves to be a promising tool in tracking community-level viral spread, the public health communication challenge lies in engaging and educating the public about the relevance and necessity of this surveillance method. A nationwide survey indicated that survey respondents had limited knowledge of the basic functions of wastewater monitoring [12]. Findings from another survey revealed a similar gap in understanding, with 49% of respondents reporting that they did not know COVID-19 can be detected in municipal sewer systems [6]. Moreover, some participants expressed skepticism and mistrust toward the method due to a lack of familiarity in the same survey. Although more recent survey data suggest overall public support for wastewater monitoring, researchers continue to call for expanded public education, as concerns regarding privacy and anonymity persist [11]. Successfully addressing these challenges involves not only gaining public support and sustaining their interest in wastewater monitoring but also encouraging individuals to take preventive measures in response to wastewater data. To this end, we investigate the message features and underlying mechanisms that influence support toward COVID-19 wastewater monitoring, interpersonal communication about the COVID-19 wastewater monitoring, and intention to take preventive measures in response to wastewater monitoring data.

### Emotional Appeals Using Different Types of Pronouns (We-Language vs You-Language)

The COVID-19 pandemic stirred widespread feelings of fear within the public, a negatively valenced emotion instigated by an emerging threat or perceived danger [13,14]. Fear has been recognized as a significant driving force behind preventive health behaviors against COVID-19, including social distancing, frequent hand washing, and mask-wearing [15,16]. Messages that evoke fear (ie, fear appeals) effectively induce high perceived severity and susceptibility, motivating individuals to adopt risk preventive measures [17]. During the pandemic, health messages often used fear appeals to promote public health behaviors [18]. Meanwhile, increasing attention has been drawn to the use of positively valenced emotions such as hope in persuasive communication (eg, [19]). Especially in the context of COVID-19, several researchers have called for a need to counterbalance prevalent negative emotions with positive emotional appeals to reduce undesired consequences, such as message avoidance and reactance [20,21]. For instance, hope plays a crucial role in aiding individuals to cope and persevere, particularly under stressful and challenging circumstances [22]. Hope appeals have been noted as a powerful motivator for behaviors that enable oneself to obtain future rewards and avoid punishment [23].

In addition to evoking emotional responses such as fear and hope, COVID-19 has triggered a sense of both individualistic and collectivistic perspectives to address the public health issue. Individually, people were confronted with risks of infection and pain from short- and long-term post symptoms even after recovery, such as a change in smell or taste, shortness of breath, chronic chest pain, depression, and changes in menstrual cycles [24]. COVID-19 was a life-threatening problem to some individuals in the most extreme cases, as more than 1 million Americans have lost their lives due to COVID-19 [25]. Simultaneously, the pandemic necessitated a communal approach, with fundamental prevention measures like masking and quarantine relying on individuals being socially aware that their actions impact not just themselves, but others in their community who might be more vulnerable [26].

Noting the dual nature of COVID-19, researchers examined the pronoun usage (ie, we vs you) to understand effective message design for combating COVID-19 [27,28]. Previous research generally showed that we-language is effective in inducing a collective self-concept, enhancing communal coping perceptions, attitudes, and social connectedness [29,30]. In the COVID-19 context, Jordan et al [27] conducted experimental studies with messages that emphasized personal, public, or their combined benefits of prevention behaviors, showing the importance of highlighting the public benefits of COVID-19 prevention behaviors.

Pronoun usage has often been investigated along with emotions as they yield some interactive influences on communicative, relational, and behavioral outcomes [31,32]. In this study, we examine the interactive effects of pronoun usage with 2 message-induced emotions, fear and hope, on attitudinal, communicative, and behavioral intentions related to COVID-19 wastewater surveillance. Given the lack of clear directional predictions in the existing literature regarding the interaction between pronoun usage and message-induced emotions, we explore these effects through research questions. Therefore, we pose the following research questions:

- RQ1: Will the type of pronouns (we-language vs you-language) used in the message moderate the influence of fear appeal on (1) support for wastewater surveillance, (2) interpersonal communication about wastewater surveillance, and (3) intention to perform COVID-19 preventive behavior?
- RQ2: Will the type of pronouns (we-language vs you-language) used in the message moderate the influence of hope appeal on (1) support for wastewater surveillance, (2) interpersonal communication about wastewater surveillance, and (3) intention to perform COVID-19 preventive behavior?

### Message Fatigue and Message Shocking Value as Mediators

To understand the mechanisms of how fear and hope appeals influence the public’s support for and understanding of wastewater monitoring against COVID-19, we considered 2 message judgments as mediators in the relationship: message fatigue and message shocking value. Message fatigue refers to an aversive motivational state caused by overexposure to campaign messages or information perceived to be similar and redundant over an extended period of time [33,34]. Message fatigue is associated with adverse cognitive, affective, and behavioral outcomes of persuasive messages, including message avoidance, annoyance, desensitization, counterargument, and less message elaboration [34].

Research suggests that emotional responses can shape the degree of message fatigue audiences experience. For instance, COVID-19 message fatigue was positively correlated with negative emotions including fear and sadness, while hope acted as a buffer against the aforementioned negative consequences [33]. We may infer that fear heightens perceived threat salience [35,36] and makes repeated exposure more taxing, thereby exacerbating message fatigue. In contrast, hope fosters a sense of efficacy and solution-oriented thinking [23], functioning as a buffer against the aversive effects of repeated exposure. Drawing on these insights, we hypothesize that COVID-19–related fear will be positively associated with message fatigue, whereas COVID-19–related hope will be negatively associated with message fatigue.

In various health contexts including COVID-19, message fatigue has been associated with reactance, yielding undesirable health outcomes such as less favorable attitudes and behavioral intentions toward positive health behaviors [37,38]. Feeling fatigued with messages has also reduced the likelihood that one will share the message content with others [39]. Despite the relatively novel aspects of wastewater monitoring, we expected to observe negative effects of message fatigue in the current study due to the general fatigue that the public is experiencing regarding COVID-19 [40]. Drawing from the previous empirical findings, we posit the following hypotheses regarding the antecedents and consequences of COVID-19 message fatigue:

- H1: Message fatigue is (1) positively associated with fear but (2) negatively associated with hope.
- H2: Message fatigue is negatively associated with (1) support for COVID-19 monitoring in wastewater, (2) intentions to talk about the monitoring with others, and (3) intentions to engage in COVID-19 safety behaviors.

We posed a message with shocking value as a mediator between the 2 discrete emotions and the proposed message outcomes. Defined as a combination of surprise and extremity regarding a message, message shocking value was positively associated with the specific emotional appraisals (eg, feeling dangerous) of threat messages [41,42]. Fear, as a primary appraisal of threat [43], may heighten sensitivity to novel or extreme elements in a message [44], thereby increasing its perceived shocking value. Hope is often considered a core element of secondary appraisals of fear that provides people with abilities and motives to take appropriate actions to escape from the current negative state [16]. In the context of wastewater monitoring, which is a relatively novel public health strategy, hope may lead individuals to interpret the message’s innovative aspects as surprising and promising, thus elevating shocking value. From the above theoretical assumptions, we expect that both fear and hope would be positively correlated with message shock value. In addition, shocking health messages are more likely to attract attention, be talked about, and be shared among people [45,46]. In line with previous findings, we expect that message shocking value will be associated with greater intentions to talk about the message topic.

However, there is a lack of empirical investigation on how message shocking value relates to behavioral outcomes, such as support for wastewater surveillance policies and engagement in preventive health behaviors. We thus posed a research question to explore the relationship between message shocking value and behavioral outcomes:

- H3: Perceived message shocking value is positively associated with (1) fear and (2) hope.
- H4: Perceived message shocking value is positively associated with the intention to talk about COVID-19 monitoring in wastewater.
- RQ3: Does perceived message shocking value have a significant association with (1) support for COVID-19 monitoring in wastewater and (2) intentions to engage in COVID-19 safety behaviors?

Additionally, as we only focused on 2 mediators in this experimental study, we hoped to explore other factors that might influence individuals’ support for waste monitoring for COVID-19 prevention. Given the exploratory nature of this inquiry and the absence of clear theoretical or empirical guidance regarding these additional factors, a research question was most appropriate. Therefore, we posed the following research question and examined the participants’ open-ended responses to the question:

- RQ4: Why do people decide to support (or not support) COVID-19 monitoring in wastewater?

## Methods

### Study Design and Message Stimuli

The study was a 2 (emotional appeal: fear appeal vs hope appeal) × 2 (types of pronouns: you-language vs we-language) between-subjects design with 1 control condition (ie, informational message only). All participants read an informative message about COVID-19 monitoring in wastewater, followed by 5 different message stimuli (see Multimedia Appendix 1 for the messages).

### Participants

Upon receiving approval from the university’s institutional review board, participants were recruited using Prolific (Prolific Academic Ltd), a widely used online crowdsourcing platform that allows researchers to post study advertisements that participants voluntarily complete in exchange for monetary compensation [47]. The present study was posted on the Prolific platform with predefined eligibility criteria, which restricted participation to US residents who are 18 years or older.

### Ethical Considerations

This study received approval from the Institutional Review Board at the University of Denver (IRB approval 2061013‐1). All participants provided informed consent for their participation and the publication of their anonymized data. The survey collected sociodemographic data (age, gender, education, race, employment, and income) but no personal identifiable information. Participants received monetary compensation (US $3.50) for completing the survey.

### Procedures

The survey was conducted between July 18 and July 19, 2023. After giving consent, participants were asked to select the wastewater use service area that matches their residential area or the one that they think is the closest to where they live. All participants then read an informative message about COVID-19 monitoring in wastewater. Participants in all 5 conditions read additional message stimuli, which were followed by questions about their emotional responses, message judgments, and attitudinal, communicative, and behavioral outcomes regarding wastewater monitoring for COVID-19.

### Measures

#### Overview

We used multi-item scales to assess fear, hope, perceived message shocking value, message fatigue, support for COVID-19 monitoring in wastewater, interpersonal communication about COVID-19 monitoring in wastewater, and preventive behaviors against COVID-19 (see Multimedia Appendix 1 for the full list of items). All variables were based on 5-point scales from 1 (*strongly disagree*) to 5 (*strongly agree*) and averaged, unless otherwise stated. Across items, there were a total of 17 missing observations. The missing data were replaced with mean scores for nonmissing observations for the given scale [48]. A confirmatory factor analysis was conducted in AMOS version 26 (Prolific Academic Ltd) to test the measurement properties of these scales. We used the following criteria to evaluate the overall model fit: *χ*^2^/*df*<3.00, comparative fit index (CFI) greater than 0.90, and root mean squared error of approximation (RMSEA) less than 0.08 [49,50]. The measurement model included all items for each latent construct, and latent constructs were allowed to covary. The fit for the initial measurement model showed room for improvement (*χ*^2^/*df*=4.51, CFI=0.91, and RMSEA=0.09, 90% CI 0.081-0.089). Based on the modification indices, we allowed the error terms of 3 pairs of items for message fatigue and 3 pairs of items for COVID-19 preventive behaviors to covary. The adjusted measurement model showed a good fit to the data (*χ*^2^/*df*=2.55, CFI=0.96, and RMSEA=0.06, 90% CI 0.053-0.061).

#### Message Informativeness

To ensure the experimental conditions do not differ in informational content, message informativeness was measured with 3 items, including “I learned something from reading the message” and “The message provided me with meaningful information” (mean 3.90, SD 0.88; *α*=.87).

#### Fear

Fear was measured by asking participants how much of the following 3 emotions—fear, scared, and afraid—they felt after reading the message. Responses, marked on the 5-point scales from 1 (*none of this emotion*) to 5 (a *great deal of this emotion*), were averaged into 1 score, with higher scores indicating greater fear (mean 1.52, SD 0.82; *α*=.96).

#### Hope

Hope was measured by asking participants how much of the following 3 emotions—hope, inspired, and encouraged—they felt after reading the message. Responses, marked on the 5-point scales from 1 (*none of this emotion*) to 5 (a *great deal of this emotion*), were averaged into 1 score, with higher scores suggesting stronger hope (mean 1.96, SD 1.14; *α*=.93).

#### Message Fatigue

Adapted from So et al [34], message fatigue was assessed with 8 items, including “Messages about COVID-19 are all beginning to sound the same to me,” and “I’m tired of hearing about the importance of COVID-19 preventive behaviors” (mean 2.95, SD 1.23; *α*=.96).

#### Message Shocking Value

Adapted from Smith et al [41], message shocking value was assessed with 3 items that measured the degree to which participants judged the message to be surprising, shocking, and alarming (mean 1.72, SD 0.79; *α*=.87).

#### Support for COVID-19 Monitoring in Wastewater

Support for COVID-19 monitoring in wastewater was assessed with 4 items including “I’m supportive of COVID-19 monitoring in wastewater” and “I think COVID-19 monitoring in wastewater is helpful” (mean 4.04, SD 1.06, *α*=.98).

#### Interpersonal Communication About COVID-19 Monitoring in Wastewater

Interpersonal communication about COVID-19 monitoring in wastewater was assessed with 5 items such as “I will tell others about COVID-19 monitoring in wastewater” and “I will talk about COVID-19 monitoring in wastewater with my family” (mean 2.81, SD 1.20; *α*=.96).

#### Intention for Preventive Behavior Against COVID-19

Intention for preventive behaviors against COVID-19 was assessed with 6 items including “I will wear a mask in indoor spaces,” and “I will try to avoid crowded locations and mass gatherings” (mean 4.14, SD 0.93; *α*=.89).

#### COVID-19 Involvement

COVID-19 involvement was assessed with 3 items: “Preventing COVID-19 is important to me,” “Preventing COVID-19 is relevant to me,” and “Preventing COVID-19 is meaningful to me” (mean 4.32, SD 0.94; *α*=.95).

#### Political Ideology

Political ideology was measured by asking participants to indicate their political ideology on a 5-point scale from 1 (*extremely liberal*) to 5 (*extremely conservative*); mean 2.36, SD 1.15.

#### Attitude Toward the Policy

Respondents’ open-ended responses were collected for an in-depth understanding of the reasons behind their support (or nonsupport) toward wastewater monitoring (RQ4). The codebook was developed after the initial review of the entire set of open-ended responses. Regarding the attitude toward the policy, the responses were categorized into support, conditional support, no support, and neutral. The reasoning category for policy support included informative value, effectiveness, novelty, nonintrusiveness, community protection, etc. The reasoning category for not supporting the policy included waste of money, government mistrust, not enough information, low perceived threat toward COVID-19, and privacy concerns. These categories were nonexclusive. The unit of analysis was a complete answer about participants’ opinions with or without specific reasons. Based on the codebook, the authors coded 20% (120/576) of the open-ended responses and resolved a few disagreements through discussion. The 2 coders independently coded the rest of the data. Intercoder reliabilities ranged from 0.94 to 1.00.

## Results

### Participants

Of the total 609 participants, 5 participants who failed the attention check question and 1 participant who took less than 5 minutes to complete the survey were eliminated from the following analyses. The final sample included 603 (n=330, 54.7% female) participants who, on average, were 39.59 (SD 12.94; median 37, min 18, max 81) years old. The average completion time was approximately 20 minutes (mean 20.38, SD 9.90 minutes). Table 1 presents demographic information of the participants.

**Table 1.** Respondent demographics.

	Frequency, n (%)
Gender	
Female	330 (54.7)
Male	261 (43.3)
Transgender	5 (0.8)
Prefer not to answer	7 (1.2)
Education	
Attended high school	10 (1.7)
Graduated from high school	209 (34.7)
Graduated from college with an associate’s degree (AA, AS, etc)	70 (11.6)
Graduated from college with a bachelor’s degree (BA, BS, etc)	233 (38.7)
Graduated with a graduate degree (MA, JD, MD, PhD, and etc)	80 (13.3)
Race^a^	
White	441 (73.1)
Black or African American	91 (15.1)
Hispanic, Latino, or Spanish origin	59 (9.8)
Asian	53 (8.8)
American Indian or Alaska Native	16 (2.7)
Native Hawaiian or other Pacific Islander	4 (0.7)
Some other race, ethnicity, or origin	5 (0.0)
Income (US $)	
<20,000	91 (15.1)
20,000-34,999	95 (15.8)
35,000-44,999	117 (19.5)
50,000-74,999	122 (20.3)
75,000 -99,999	74 (12.3)
≥100,000	101 (16.8)
Employment	
Employed by a company that you do not own	334 (55.4)
Self-employed	95 (15.8)
Full-time student	36 (6.0)
Unemployed	97 (16.1)
Retired	41 (6.8)

a Participants were allowed to respond to multiple options for race/ethnicity.

### Manipulation Checks

We first assessed whether (1) the fear appeal was the most fear-inducing, (2) the hope appeal was the most hope-inducing, and (3) the 5 messages were comparable in levels of informativeness. The ANOVA results showed that participants reported greater fear in response to the fear appeal (mean 1.73, SD 0.91, 95% CI 1.62‐1.85) than the hope appeal (mean 1.40, SD 0.72, 95% CI 1.31‐1.49) or informational appeal (mean 1.36, SD 0.72, 95% CI 1.23‐1.49; *F*_2,600_*=*13.59; *P*<.001; η^2^=0.04). The ANOVA results showed that participants reported greater hope in response to the hope appeal (mean 2.10, SD 1.24, 95% CI 1.95‐2.26) than the fear appeal (mean 1.84, SD 1.08, 95% CI 1.70‐1.98), but there was no statistically significant difference with the informational appeal (mean 1.93, SD 1.03, 95% CI 1.75‐2.12; *F*_2,600_*=*3.27; *P*=.04; η^2^=0.01). The ANOVA results showed that perceived informativeness did not vary by message condition (*F*_4,598_*=*1.98; *P*=.10; η^2^=0.01). The CIs for the message conditions overlapped, indicating that the difference between the conditions was not statistically significant: informational appeal (mean 4.06, SD 0.67, 95% CI 3.94‐4.18), fear appeal in we-language (mean 4.03, SD 0.88, 95% CI 3.87‐4.18), hope appeal in we-language (mean 3.95, SD 0.80, 95% CI 3.81‐4.10), fear appeal in you-language (mean 3.85, SD 0.91, 95% CI 3.69‐4.01), and hope appeal in you-language (mean 3.81, SD 0.93, 95% CI 3.65‐3.98). In the subsequent analyses, we included a total of 482 participants in the fear appeal and hope appeal conditions.

### Preliminary Analyses

To inspect overall patterns in the data, we examined the means and SDs of all variables and the correlations among them. Table 2 presents the correlation matrix.

**Table 2.** Correlation matrix for the study variables (n=603)^a^.

	1	2	3	4	5	6	7	8	9
(1) Fear									
* r*	1								
*P* value	—^b^								
(2) Hope									
* r*	0.06	1							
*P* value	.17	—							
(3) Msgfatig									
* r*	–0.18	–0.32	1						
*P* value	<.001	<.001	—						
(4) Msgshock									
* r*	0.53	0.12	–0.12	1					
*P* value	<.001	.002	.003	—					
(5) Support									
* r*	0.10	0.35	–0.51	0.13	1				
*P* value	.01	<.001	<.001	<.001	—				
(6) Comm									
* r*	0.26	0.34	–0.42	0.29	0.45	1			
*P* value	<.001	<.001	<.001	<.001	<.001	—			
(7) Intention									
* r*	0.11	0.21	–0.49	0.10	0.63	0.37	1		
*P* value	.005	<.001	<.001	.01	<.001	<.001	—		
(8) Involve									
* r*	0.17	0.24	–0.51	0.14	0.59	0.38	0.67	1	
*P* value	<.001	<.001	<.001	<.001	<.001	<.001	<.001	—	
(9) Political									
* r*	–0.04	–0.05	0.37	0.04	–0.37	–0.16	–0.36	–0.30	1
*P* value	.36	.19	<.001	.33	<.001	<.001	<.001	<.001	—

a Msgfatig refers to message fatigue, msgshock refers to message shocking value, support refers to support for wastewater surveillance, comm refers to interpersonal communication about wastewater surveillance, intention refers to intention to perform COVID-19 preventive behavior, involve refers to involvement with COVID-19, and political refers to political ideology (ie, higher score refers to stronger conservative political ideology).

b Not applicable.

One notable feature of the data was relatively low mean values for fear and hope. We also noted the relatively high levels of support for COVID-19 monitoring in wastewater and intentions to engage in COVID-19 safety behaviors. Because COVID-19 involvement and political ideology showed significant associations with substantive variables, we conducted our main analyses with these variables as covariates, following the procedure by Tabachnick and Fidell [51].

### Hypotheses and RQs

#### Emotional Appeals and Pronoun Use Across Message Outcomes

To explore RQ1 and RQ2, we ran a series of 2 (emotional appeal: hope vs fear appeals) × 2 (pronouns: we- vs you-language) analysis of covariance to examine message effects on support for COVID-19 wastewater monitoring, interpersonal communication about COVID-19 wastewater monitoring, and COVID-19 preventive behavior intention, controlling for involvement with COVID-19 and political ideology. Results of a 2-way analysis of covariance found that fear appeal (mean 4.11, SD 1.00) led to significantly higher support for COVID-19 wastewater monitoring than hope appeal (mean 3.91, SD 1.16; *F*_1,476_*=*5.15; *P*=.02; partial *η*^2^=0.011). Pronouns used in the message (*F*_1,476_*=*1.57; *P*=.21; partial *η*^2^=0.003) or interaction between emotional appeal and pronouns (*F*_1,476_*=*1.83; *P*=.18; partial *η*^2^=0.004) did not influence higher support for COVID-19 wastewater monitoring. Neither the main effects of emotional appeal (*F*_1,476_=0.53; *P*=.47; partial *η*^2^=0.001) and pronouns (*F*_1,476_=0.03; *P*=.86; partial *η*^2^=0.001) nor the interaction effects of the two (*F*_1,476_=0.75; *P*=.39; partial *η*^2^=0.002) were significant for interpersonal communication about COVID-19 wastewater monitoring. Fear appeal (mean 4.18, SD 0.87) led to significantly higher intentions for COVID-19 preventive behavior than hope appeal (mean 4.02, SD 1.03; *F*_1,476_=4.67; *P*=.03; partial *η*^2^=0.01). Pronouns used in the message (*F*_1,476_*=*1.30; *P*=.26; partial *η*^2^=0.003) or interaction between emotional appeal and pronouns (*F*_1,476_=0.03; *P*=.86; partial *η*^2^=0.000) did not influence higher intentions for COVID-19 preventive behavior.

#### Indirect Mechanisms of Message Fatigue and Shocking Value

**Figure 1.**
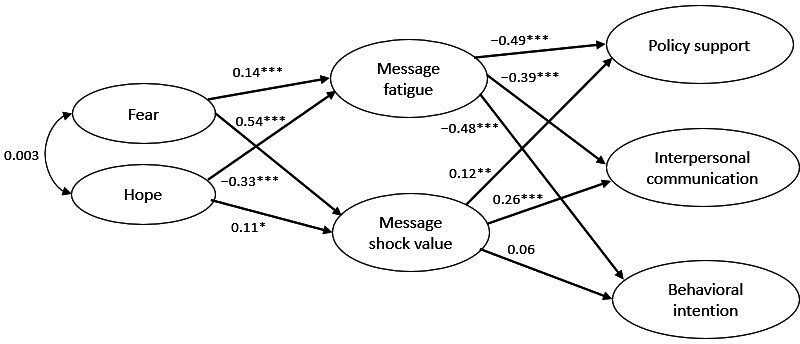
Structural equation modeling (SEM) diagram presenting the effects of discrete emotions on the 3 outcome variables mediated by message fatigue and perceived message shocking value. Note: The model presents standardized regression weights. Covariate and error terms were included in the analysis and are omitted from the figure for the sake of parsimony. *P<.05; **P<.01; ***P<.001.

Hypotheses H1-H4 and RQ3 were tested using structural equation modeling. The initial model included 2 exogenous variables (ie, fear and hope). Perceived message shocking value and message fatigue were treated as mediating variables, and support for COVID-19 monitoring in wastewater, interpersonal communication about COVID-19 monitoring in wastewater, and preventive behaviors against COVID-19 were the outcome variables. The initial model included all possible paths from the exogenous variables to the mediating variable, all possible paths from the mediating variable to the outcome variables, and no direct paths from the exogenous variables to the outcome variables. COVID-19 involvement and political ideology were added as covariates to the model. The estimated model indicated good fit to the data (*χ*^2^/*df*=2.70, CFI=0.95, and RMSEA=0.06, 90% CI 0.056-0.063). Figure 1 presents the significant pathways between the exogenous and endogenous variables.

H1 predicted (1) a positive relationship between fear and message fatigue while proposing (2) a negative relationship between hope and message fatigue. Both fear (*β*=–0.21; *P*<.001) and hope (*β*=–0.32; *P*<.001) were negatively associated with message fatigue, partially supporting H1.

H2 proposed a negative association between message fatigue and the 3 proposed outcomes. Supporting H2, message fatigue was negatively associated with (1) support for COVID-19 monitoring in wastewater (*β*=–0.53; *P*<.001), (2) intentions to talk about the monitoring with others (*β*=–0.41; *P*<.001), and (3) intentions to engage in COVID-19 safety behaviors (*β*=–0.37; *P*<.001).

H3 proposed a positive relationship (1) between the message shocking value and fear, and (2) between the message shocking value and hope. Supporting H3, both fear (*β*=0.61; *P*<.001) and hope (*β*=0.08; *P*<.01) were positively associated with perceived message shocking value (*β*=0.61; *P*<.001).

H4 and RQ3 were proposed to examine the relationship between the perceived message shocking value and the 3 proposed outcomes. Supporting H4, the message shocking value was positively associated with intentions to talk about COVID-19 wastewater monitoring with others (*β*=0.34; *P*<.001). Regarding RQ3, the results showed that the perceived message shocking value was positively associated with support for COVID-19 monitoring in wastewater (*β*=0.13; *P*<.01) but had a nonsignificant relationship with intentions to engage in COVID-19 safety behaviors (*β*=0.07; *P*=.15).

#### Themes From Open-Ended Responses on Reasons for Supporting Wastewater Monitoring

Participants’ open-ended responses allowed for a more in-depth understanding of their attitude toward wastewater monitoring (RQ4). Most respondents (n=425, 74.2%) indicated they supported COVID-19 monitoring in wastewater, whereas 62 (10.8%) did not support the policy and 61 (10.6%) answered as neutral. For reasons to support wastewater monitoring, most of the respondents claimed that they support the intervention considering its effectiveness and informative value, followed by offering protection to the community. The leading reason for not supporting the intervention was due to financial concerns, followed by a low-risk perception of COVID-19 and government mistrust. A total of 19 respondents who were neutral about the intervention stated that they would need more information before forming an opinion, while others did not specify reasons for their neutral stance. The coding categories and examples for each category are presented in Table 3.

**Table 3.** Examples, frequency, and intercoder reliability of the reasons for supporting and not supporting the COVID-19 wastewater monitoring policy.

Coding category	Examples	Frequency, n	Intercoder reliability, k
Reasons for supporting COVID-19 wastewater monitoring			
Informative value	“I support any means to keep track of the outbreaks.”	138	1.00
Effectiveness	“I do support it. It is another tool we can use to stop infections.”	157	0.94
Novelty	“I would support it because it’s an effective, new technique that can beneficially help communities know about outbreaks quickly.”	2	1.00
Nonintrusiveness	“Sure, because it’s a non-invasive way to track COVID spread.”	37	1.00
Increase public awareness	“I think we should because it’s a potential public health hazard regardless.”	13	1.00
For vulnerable population	“I would support it because I think it’s important to know if there is a spike of COVID in a community, especially for those who are at risk.”	1	1.00
Protect community	“I support any effort done to monitor and minimize COVID-19 in the community.”	96	0.96
Importance of COVID-19	“Yes I support it. If it helps eradicate COVID; I’m all for it.”	36	0.96
No downside	“I don’t see any reason to not support it.”	29	1.00
Reasons for not supporting COVID-19 wastewater monitoring			
Waste of resources	“I wouldn’t support it. It’s a waste of money and resources.”	19	1.00
Government mistrust	“Not a big deal. Gov makes big deal out of nothing to control and gain power.”	10	1.00
Not enough information	“I would not support the COVID-19 monitoring in wastewater until I knew it was real.”	5	0.96
Low perceived threat	“COVID has a low fatality rate and is no longer important.”	12	0.96
Privacy concerns	“I do not support monitoring wastewater because I believe it is an invasion of citizen’s privacy.”	5	1.00

## Discussion

### Principal Findings

This study investigated the impact of emotional appeals (ie, fear and hope appeals) and pronoun use on the attitudinal, communicative, and behavioral outcomes related to wastewater monitoring for COVID-19. Although the main effect of pronoun use was not significant, we observed that the fear appeal led to greater support for the policy and intentions to engage in COVID-19 preventive behaviors compared to the hope appeal. Additionally, significant mediating mechanisms, involving message fatigue and shock value, were observed between message-evoked emotions (ie, fear and hope) and the proposed outcomes.

While empirical data reveal the impact of pronoun use on people’s emotions and judgments, we did not observe the main effect of pronoun use on our proposed outcomes. We speculate that testing the psycholinguistic features (specifically “we” vs “you”) alongside emotional appeals may have confounded the effects. “You” language is often associated with negative feelings such as fear, especially when the source of a problem is being investigated. This attributive nature in “you” language facilitates denigrating or accusing a person of wrongdoing (eg, you should have behaved this way to prevent COVID-19; [52]). Conversely, “we” language obscures the singular source of the problem, making it less explicit. Thus, using “you” language in messages can effectively induce fear, whereas fear appeals in “we” language are more prone to trigger cognitive dissonance by conveying contradictory perceptions. Future research should delve into the distinct emotional valence associated with each pronoun.

We found intriguing mediating mechanisms between discrete emotions and COVID-19-related preventive measures using message fatigue and message shocking value as mediators. Both fear and hope were negatively correlated with message fatigue, with hope acting as a buffer, aligning with previous research [53]. The unexpected negative relationship between fear and message fatigue may be related to fear’s novelty-inducing nature [54]. Given the prevalent message fatigue around COVID-19, experiencing any emotion might reduce fatigue. Participants with lower message fatigue were more likely to support COVID-19 wastewater monitoring, engage in discussions, and adopt preventive behaviors drawn from wastewater data. This suggests that emotional appeals may be particularly effective when addressing issues with high message fatigue.

As predicted, both fear and hope were positively associated with message shocking value, supporting the notion that messages that evoke discrete emotions are likely to be perceived as shocking [55]. The results highlight the importance of message shocking value as the mediating mechanism, as shocking messages promote support toward the novel health intervention and encourage intentions for discussions. Meanwhile, the message shocking value did not have a significant influence on preventive actions against COVID-19, as the message shocking value was mostly driven by the description of the novel health intervention in the message, rather than the information about COVID-19 preventive actions that were also presented in the message. Future research should explore ways to translate public interest in novel health interventions into adherence to recommended health behaviors.

Practically, we believe that the use of emotions and different pronouns in message design can be applied to other contexts and is worth investigating further, as they can generate unique psycholinguistic features in the messages. Although we did not find an interaction effect between the 2 message features, we believe that this is one of the crucial areas of message design that can be applicable to various contexts in need of more attention. More importantly, our results regarding the effectiveness of emotional appeals as a remedy for message fatigue demonstrate that well-designed messages can counteract fatigue in topics that typically suffer from audience disengagement, such as COVID-19 or climate change, thereby sustaining attention and support for diverse public health and environmental issues. This potential to alleviate message fatigue represents a central contribution of the present study.

Furthermore, this was one of the first studies to investigate effective message strategies to increase public support and understanding of COVID-19 wastewater monitoring. Analysis of the open-ended responses revealed that support for this intervention is highly affected by the perceived effectiveness and informative value. To garner public support, emphasizing the unique advantages and efficiency of COVID-19 wastewater monitoring compared to other interventions is crucial. Moreover, concerns about wasting resources and government mistrust drew people away from favoring the policy. While establishing trust in government requires long-term sustained efforts, public health campaigns can effectively focus on alleviating concerns related to resource wastage. Health messages to increase public support toward wastewater monitoring of COVID-19 could, for instance, highlight the cost-effective aspect of this intervention. Considering several participants who were neutral about the intervention due to their desire for more information before forming an opinion, it seems especially pertinent to underscore in the health messages not only the necessity of COVID-19 wastewater monitoring but also its functionality in enhancing public health.

### Limitations

This study is not without limitations. First, this study used a cross-sectional design, which makes it difficult to draw causal inferences. Future research should use longitudinal designs or repeated-exposure experiments to determine if these emotional appeals can effectively mitigate “message fatigue” over the course of an extended period. Next, the generalizability of these findings may be limited by the specific context of the study. COVID-19 wastewater monitoring represented a novel health issue by the time of data collection, and it is possible that the message effects observed in the current study context may differ when applied to more routine health behaviors through different mechanisms. Future research should investigate whether message strategies in this study hold true for diverse health topics. In addition, our use of an online convenience sample may further limit the generalizability of the findings. Future research should examine these relationships using more diverse and representative samples. We also note that the context of our study can be referred to as being paradoxical, in which the situational setting of message topic was concerned with feelings of fatigue and boredom (ie, overwhelming messages about COVID-19 prevention) and feelings of novelty (ie, wastewater monitoring as a novel health intervention) simultaneously. While this setting provided us with an interesting context to test the predictions, we wondered if such a context had any influence on the message effects. This remains speculative due to the lack of empirical data in the current study, leaving room for future investigation. At last, although several effects were statistically significant, their magnitudes were small, which limits conclusions regarding the practical or real-world significance of findings. Future research should examine whether small effects accumulate over time or translate into meaningful behavioral change in real-world public health communication contexts.

### Conclusions

In conclusion, this study investigated the effects of emotional appeals and pronoun use on the communicative, attitudinal, and behavioral consequences of messages regarding wastewater monitoring for COVID-19. The current study sheds light on understanding the influence of message-evoked emotions in the unique context of promoting the public’s support and understanding of wastewater monitoring for COVID-19 and the underlying mediating mechanisms that add to its explanatory power. By showing that lower message fatigue was associated with greater policy support, discussion intentions, and preventive behavior intentions, our findings highlight the potential for well-designed emotional appeals to counteract fatigue on high-exposure issues. This capacity to mitigate message fatigue, which is relevant not only to COVID-19 but also to other sustained public health and environmental challenges such as climate change, represents a meaningful contribution to both theory and practice.

## Supplementary material

10.2196/83060 Multimedia Appendix 1 Message stimuli and measurement items.

## Abbreviations

CFI: comparative fit index
RMSEA: root mean square error of approximation
