# Supplementary material for: The Role of Fear, Hope, Message Fatigue, and Message Shocking Value in Promoting the Public’s Understanding and Support Toward COVID-19 Wastewater Monitoring: Experimental Study
Source: JMIR Form Res. 2026 Feb 27;10:e83060. doi: 10.2196/83060 (PMC12954702; doi:10.2196/83060)
Supplement: Multimedia Appendix 1 [file formative-v10-e83060-s001.docx]

**Supplementary Files**

**Message Stimuli**

The following infographic about the background and general description of wastewater monitoring policy was presented to all conditions prior to the main message.

People infected with SARS-CoV-2 can shed the virus in their feces, even if they don’t have symptoms. The virus can then be detected in wastewater, enabling wastewater monitoring to capture the presence of SARS-CoV-2 shed by people with and without symptoms. This allows wastewater monitoring to serve as an early warning that COVID-19 is spreading in a community.

A graphic description of the wastewater monitoring process is as follows:

**
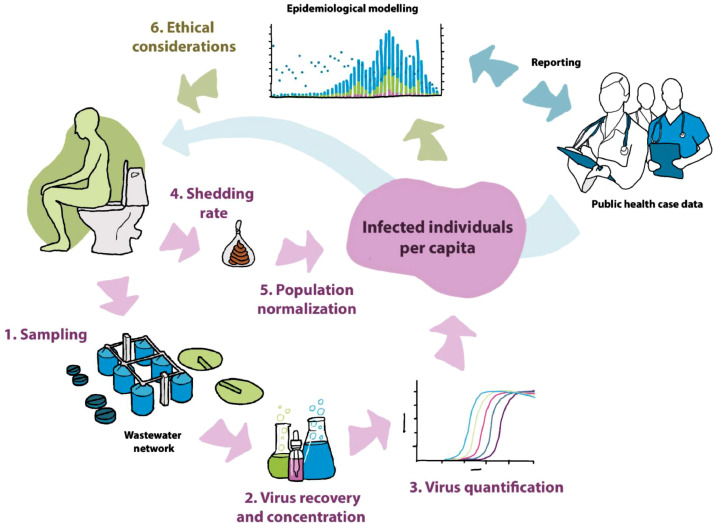
**

1. Information only condition

**Keep up with COVID-19 preventive behaviors!**

Higher levels of coronavirus in wastewater can indicate a rise in COVID-19 cases. If the community’s monitoring data shows high levels of COVID-19 virus particles, remember to keep up with COVID-19 preventive behaviors.

Preventive measures against COVID-19 include basic health and hygiene behaviors, wearing a mask in indoor spaces, and avoiding crowded locations. It is also important to monitor for symptoms and get tested for COVID-19 if needed.

2. Fear $\times$ we condition

**Do not forget the threats of COVID-19 to us!**

Know that we are facing a dire situation. We should be aware that COVID-19 is a highly severe and infectious disease. New variants of SARS-CoV-2, the virus that causes COVID-19, continue to occur, and our chances of developing the severe disease are real.

Higher levels of coronavirus in wastewater can indicate a rise in COVID-19 cases, which may lead to dangerous outcomes. If wastewater monitoring data for our community shows high levels of COVID-19 virus particles, we should be worried and keep up with COVID-19 preventive behaviors.

We still have a good chance of catching and suffering from COVID-19. To avoid a devastating future, we should keep up with basic health and hygiene behaviors, wear a mask in indoor spaces, and avoid crowded locations. We should also monitor ourselves for symptoms and get tested for COVID-19 if needed.

We can reduce fear of the COVID-19 pandemic by playing our parts.

3. Fear $\times$ you condition

**Do not forget the threats of COVID-19 to you!**

Know that you are facing a dire situation. You should be aware that COVID-19 is a highly severe and infectious disease. New variants of SARS-CoV-2, the virus that causes COVID-19, continue to occur, and your chances of developing the severe disease are real.

Higher levels of coronavirus in wastewater may indicate a rise in COVID-19 cases, which can lead to dangerous outcomes. If wastewater monitoring data for your community shows high levels of COVID-19 virus particles, you should be worried and keep up with COVID-19 preventive behaviors.

You still have a good chance of catching and suffering from COVID-19. To avoid a devastating future, you should keep up with basic health and hygiene behaviors, wear a mask in indoor spaces, and avoid crowded locations. You should also monitor yourself for symptoms and get tested for COVID-19 if needed.

You can reduce fear of the COVID-19 pandemic by playing your part.

4. Hope $\times$ we condition

**Imagine a better future for us!**

Know that a bright future is coming toward us. We should be aware that there are many things that we can do to stay safe from COVID-19. Even though new variants of SARS-CoV-2, the virus that causes COVID-19, continue to occur, we can protect ourselves to bring about a better future for us.

Higher levels of coronavirus in wastewater may indicate a rise in COVID-19 cases. Even if wastewater monitoring data for our community shows high levels of COVID-19 virus particles, we should hold on to hope and keep up with COVID-19 preventive behaviors.

We can move forward from COVID-19. To achieve a better future, we should keep up with basic health and hygiene behaviors, wear a mask in indoor spaces, and avoid crowded locations. We should also monitor ourselves for symptoms and get tested for COVID-19 if needed.

We can increase hope for ending the COVID-19 pandemic by playing our parts.

5. Hope $\times$ you condition

**Imagine a better future for yourself!**

Know that a bright future is coming toward you. You should be aware that there are many things that you can do to stay safe from COVID-19. Even though new variants of SARS-CoV-2, the virus that causes COVID-19 continue to occur, you can protect yourself to bring about a better future for you.

Higher levels of coronavirus in wastewater may indicate a rise in COVID-19 cases. Even if wastewater monitoring data of your community shows high levels of COVID-19 virus particles, you should hold on to hope and keep up with COVID-19 preventive behaviors.

You can move forward from COVID-19. To achieve a better future, you should keep up with basic health and hygiene behaviors, wear a mask in indoor spaces, and avoid crowded locations. You should also monitor yourself for symptoms and get tested for COVID-19 if needed.

You can increase hope for ending the COVID-19 pandemic by playing your part.

**Measurement Items**

**Message Informativeness (3 items)**

First, we would like to know your impressions of the message. Please indicate the extent to which you agree or disagree with each statement (*1 = Strongly disagree, 2 = disagree, 3 = neither disagree nor agree, 4 = agree, 5 = strongly agree*).

- I learned something from reading the message.
- The message was informative.
- The message provided me with meaningful information.

**Message Fatigue (8 items)**

Please indicate the extent to which you agree or disagree with each statement *(1 = Strongly disagree, 2 = disagree, 3 = neither disagree nor agree, 4 = agree, 5 = strongly agree).*

- At this point, I’ve heard about problems related to COVID-19 more than I ever needed to.
- There are simply too many messages about COVID-19 nowadays.
- After hearing them for years, messages about COVID-19 seem repetitive.
- Messages about COVID-19 are all beginning to sound the same to me.
- I’m sick of hearing about problems associated with COVID-19.
- I’m tired of hearing about the importance of COVID-19 preventive behaviors.
- I find messages about COVID-19 to be dull and monotonous.
- Messages about COVID-19 are tedious.

**Message Shocking Value (3 items)**

How did the message make you feel? (*1 = None of this emotion, 2 = a little bit, 3 = somewhat, 4 = moderate, 5 = a great deal of this emotion*).

- Shocking
- Surprising
- Alarming

**Support for COVID-19 Monitoring in Wastewater (4 items)**

Please indicate the extent to which you agree or disagree with each statement (*1 = Strongly disagree, 2 = disagree, 3 = neither disagree nor agree, 4 = agree, 5 = strongly agree*).

- I am supportive of COVID-19 monitoring in wastewater.
- I think COVID-19 monitoring in wastewater is helpful.
- I think COVID-19 monitoring in wastewater is useful.
- I support the decision to monitor COVID-19 in wastewater.

**Interpersonal Communication about COVID-19 Monitoring in Wastewater (5 items)**

Please indicate the likelihood that you would like to share the information about COVID-19 monitoring data (*1 = Very unlikely, 2 = unlikely, 3 = unsure, 4 = likely, 5 = very likely*).

- I will tell others about COVID-19 monitoring in wastewater.
- I will share information about COVID-19 monitoring in wastewater with others.
- I will talk about COVID-19 monitoring in wastewater with my family.
- I will talk about COVID-19 monitoring in wastewater with my friends.
- I will talk about COVID-19 monitoring in wastewater with people living in my residential area.

**Intention for Preventive Behavior against COVID-19 (6 items)**

With these questions, we would like to know your intention for COVID-19 preventive behaviors upon viewing the wastewater monitoring data (*1 = Very unlikely, 2 = unlikely, 3 = unsure, 4 = likely, 5 = very likely*)*.*

If wastewater monitoring data for my residential area shows high levels of COVID-19 virus particles,

- I will wear a mask in indoor spaces.
- I will try to avoid crowded locations and mass gatherings.
- I will wash or sanitize my hands more frequently.
- I will avoid contact with people who have suspected or confirmed COVID-19.
- I will follow recommendations for isolation if you have suspected or confirmed COVID-19.
- I will get tested if I have COVID-19 symptoms.

**COVID-19 Involvement**

The next question relates to your understanding of and experience with COVID-19. (*1 = Strongly disagree, 2 = disagree, 3 = neither disagree nor agree, 4 = agree, 5 = strongly agree*).

- Preventing COVID-19 is important to me.
- Preventing COVID-19 is relevant to me.
- Preventing COVID-19 is meaningful to me.

**Political Ideology**

Please indicate your political ideology (*1= Extremely liberal, 2 = slightly liberal, 3 = moderate or middle of the road, 4 = slightly conservative, 5 = extremely conservative*).
